# Relationship between primary monosymptomatic enuresis and process toilet training: a case-control

**DOI:** 10.1590/S1677-5538.IBJU.2022.0381

**Published:** 2022-09-20

**Authors:** Tânia Antunes Carvalho, Mônica Maria de Almeida Vasconcelos, José de Bessa, José Murillo Bastos, Melissa Faria Dutra, Isabela Cristina de Oliveira Guimarães, Eleonora Moreira Lima, Ana Cristina Simões e Silva, Flávia Cristina de Carvalho Mrad

**Affiliations:** 1 Universidade Federal de Minas Gerais Faculdade de Medicina Departamento de Pediatria Belo Horizonte MG Brasil Departamento de Pediatria, Unidade de Nefrologia Pediátrica, Faculdade de Medicina, Universidade Federal de Minas Gerais (UFMG), Belo Horizonte, MG, Brasil; 2 Universidade Estadual de Feira de Santana Departamento de Urologia Feira de Santana BA Brasil Departamento de Urologia, Universidade Estadual de Feira de Santana (UFFS), Feira de Santana, BA, Brasil; 3 Universidade Federal de Juiz de Fora Faculdade de Medicina Departamento de Urologia Juiz de Fora MG Brasil Departamento de Urologia, Faculdade de Medicina, Universidade Federal de Juiz de Fora (UFJF), Juiz de Fora, MG, Brasil; 4 Faculdade de Ciências Médicas de Juiz de Fora Maternidade Therezinha de Jesus Departamento de Urologia Juiz de Fora MG Brasil Departamento de Urologia, Maternidade Therezinha de Jesus, Faculdade de Ciências Médicas de Juiz de Fora, Juiz de Fora, MG, Brasil; 5 Universidade Federal de Minas Gerais Laboratório Interdisciplinar de Investigação Médica Belo Horizonte MG Brasil Laboratório Interdisciplinar de Investigação Médica, Universidade Federal de Minas Gerais, Belo Horizonte, MG, Brasil

**Keywords:** Enuresis, Toilet Training, Adolescent

## Abstract

**Objective:**

Primary monosymptomatic nocturnal enuresis (PMNE) is a prevalent condition in childhood, and the pathophysiology is multifactorial. This study investigated the relationship between the toilet training process (TT) and PMNE in children and adolescents.

**Patients and Methods:**

A case-control study was carried out from 2015 to 2020. The presence of PMNE was identified according to International Children's Continence Society criteria. A semi-structured questionnaire was applied to assess TT.

**Results:**

The study included 103 children and adolescents with PMNE and 269 participants with normal psychomotor development without PMNE (control group [CG]). Readiness signals were more remembered and less frequent in participants with PMNE (p=0.001) when compared to control group. No differences were found between the groups regarding the onset age of the daytime TT (p= 0.10), the nocturnal TT (p=0.08), the acquisition of daytime continence (p=0.06), and the type of equipment used for the TT (p=0.99). The use of Child-Oriented approach in group of children with enuresis was lower than in controls [87.4% (90/103) versus 94% (250/266)], respectively (OR= 0.44, 95% CI 0.21-0.94, p = 0.039).

**Conclusions:**

The age of onset of TT, acquisition of daytime continence, and the type of equipment were not associated with higher occurrence of PMNE. On the other hand, the Child-Oriented approach was a protective factor for the occurrence of PMNE.

## INTRODUCTION

Monosymptomatic nocturnal enuresis is defined by the International Children's Continence Society (ICCS) as isolated urinary incontinence during sleep in children aged five years and older, with no associated clinical condition to justify it. Primary monosymptomatic nocturnal enuresis (PMNE) occurs in children and adolescents who have never achieved a period greater than six continuous months of nighttime dryness (1, 2). PMNE is more prevalent in male gender with a 2:1 ratio at any age and affects about 5-10% of 7-year-old children (3). The prevalence in adolescents is around 3% and from 0.5 to 1% in adults (2, 4, 5). The spontaneous remission rate is about 15% (2, 3).

PMNE often leads to loss of self-esteem, compromised school learning, and difficulties in relationships with peers and family (2, 3, 6). The etiology is multifactorial, and the main pathogenic mechanisms include nocturnal polyuria, detrusor overactivity, increased arousal threshold, and genetic predisposition (2, 7). However, the pathophysiology is not fully understood (2, 7–9). Some aspects of the toilet training (TT) process have been associated with the occurrence of PMNE (10, 11).

TT is an important milestone in child development, which can be affected by anatomical, physiological, behavioral, and cultural conditions (12, 13). Over 50 years, the average age to start TT has been delayed from 18 to 24-36 months in children with normal neuropsychomotor development. The same happened concerning the average age to complete the TT, which went from 24 to 36-39 months (13–15). On the other hand, in some Asian and African countries, very early TT is commonly used, starting from two to three weeks of age, and finishing around 12 months of age (16, 17). Girls, more frequently, start and complete the TT earlier than boys (18). The method of TT can be categorized as the Child-Oriented approach and the Structured Behavioral approach (13, 19). The American Academy of Pediatrics (AAP) recommends the Child-Oriented approach, based on Brazelton's Method (20), for children with normal neuropsychomotor development. This approach recommends the start of TT only when the child shows signs of readiness (12, 13, 20). On the other hand, the Structured Behavioral approach is guided by the parents (Azrin and Foxx Method (21), Infant Assisted Training (16), and Elimination Communication (17) and does not consider the child's need for readiness to initiate TT (19).

There are still a lot of discussion about when to start the TT, what would be the best approach and its possible repercussions on the lower urinary tract. We hypothesize that the TT process might be related to the occurrence of PMNE in children and adolescents. In this sense, the present study aimed to evaluate the relationship between the occurrence of PMNE, the age of beginning and completion of the TT, the approach, and the type of equipment used in this process.

## PATIENTS AND METHODS

### Ethical approval

The institution Ethics Committee approved the study CAAE 86171118.0.0000.514, under protocol number 2.625.013 (April 27, 2018). The legal guardians of the patients signed an informed consent form.

### Study design

This is a case-control study with prospectively collected data in which 133 children and adolescents with PMNE were initially evaluated as cases. Thirty were excluded for the following reasons: five had spina bifida occulta, ten had intellectual development disorders, one had diabetes mellitus, one had sickle cell disease and 13 had non-monosymptomatic enuresis. Therefore, the case group consisted of 103 children and adolescents with PMNE, aged between five and 12 years, who regularly attended an Enuresis Outpatient Clinic from February 2015 to February 2020. The control group (CG) consisted of 266 children and adolescents with normal neuropsychomotor development and without lower urinary tract symptoms matched by sex, age and socioeconomic status that attended a primary healthcare unit.

### Exclusion criteria

Children and adolescents with intellectual development disorder, congenital anomalies of the nervous system, urogenital malformations, presence of diseases and/or use of medications that interfere with the functioning of the bladder or urethral sphincter, diabetes, sickle cell disease, non-monosymptomatic diseases and/or enuresis secondary school or who refused to participate in the study were excluded

### Study protocol

The diagnosis of PMNE was based on the ICCS criteria, defined as urinary incontinence during sleep in children aged at least five years with at least one episode per month and a minimum duration of three months, excluding organic causes. (1, 2). Following the care protocol of the Enuresis Outpatient Clinic based on the ICCS (1, 2) and the Brazilian Consensus on Enuresis (22), guided anamnesis, urinalysis, urine culture, renal and bladder ultrasound and calculation of nighttime urinary volume were performed. In addition, a bladder and bowel diary and a calendar of dry nights were requested. The Dysfunctional Voiding Symptom Score (DVSS) adapted for this population was used to diagnose PMNE. The cutoff values to indicate the presence of lower urinary tract symptoms (LUTS) were greater than six for girls and nine for boys (23).

A semi-structured questionnaire not yet validated was developed and applied to parents to assess the TT process. The questionnaire was based on previous studies (19, 24). It included the signs of readiness, the age at which the child started and completed the TT, the approach (Child-Oriented or Structured Behavioral), and the type of equipment (potty chair, regular toilet, toilet with seat reducer, toilet with footrest, toilet with a seat reducer) used (Appendix A). TT completion was defined as the age at which the child achieved complete bowel and bladder control without failing to retain urine or stool during the day and night (14). The pediatricians were trained to apply the instruments and conducted the interviews with the subjects and their parents in a confidential environment.

#### Statistical Analysis

The software GraphPad Prism, version 9.0.3 (GraphPad Prism®, San Diego-CA, USA) was used for statistical analysis. The Shapiro Wilk test evaluated the distribution of the numerical variables. Continuous quantitative variables were expressed as means and standard deviations. Categorical variables were shown as absolute values or proportions. Student's t-test or Mann-Whitney test compared continuous variables according to distribution, whereas chi-square test was used for categorical variables comparisons. Odds Ratio (OR) with 95% confidence interval (95% CI) evaluated the magnitude and precision of the association between categorical variables. Values of p<0.05 were considered statistically significant.

## RESULTS

The case group was composed of 63.1% of male gender (65/103) with a mean age of 7.5 ± 3.11 years. Control group (CG) had a mean age of 7.3 ± 2.88 years, with 57.1% of male gender (152/266). As shown in Table-1, no significant differences were found concerning age, gender and socioeconomic status when comparing the groups.

**Table 1:** Baselines and characteristics of toilet training in children and adolescents with primary monosymptomatic nocturnal enuresis and the control group.

Characteristics		Children and adolescents with PMNE (n=103)	Control group (n=266)	p
**Gender Male**		63.1% (65/103)	57.1% (152/266)	0.09
Age mean (SD)		7.3 ± 2.88	7.5 ± 3.11	0.10
**Toilet Training Readiness**				0.001
	Present	42.7% (44/103)	54.1% (144/266)	
	Absent	40.8% (42/103)	15% (40/266)
	Not remembered	16.5% (17/103)	31% (82/266)	
**Approach**				0.03
	Child-oriented	87.4% (90/103)	94% (250/266)	
	Structural Behavior	12.6% (13/103)	7.1% (19/266)	
**Type of equipment**				0.99
	Potty Chair	65% (67/103)	66.5% (177/266)	
	Regular toilet	26.2% (27/103)	25.2% (67/266)	
	Toilet with seat reducer	4.9% (5/103)	4.5% (12/266)	
	Toilet with footrest	1.9% (2/103)	2.3% (6/266)	
	Toilet with a seat reducer and footrest	1.9% (2/103)	1.5 % (4/266)	
**Time**		Age mean (SD) months		
Started the daytime TT		18.6 ± 8.7	17.4 ± 4.9	0.10
Acquisition of daytime continence		23.2 ± 11.3	21.4 ± 6.1	0.06
Started the nighttime TT		22.3 ± 3.1	20.1 ± 5.5	0.08

PMNE = Primary monosymptomatic nocturnal enuresis; SD = Standard Deviation; TT = Toilet training.

*p* value < 0.05.

**Figure 1:**
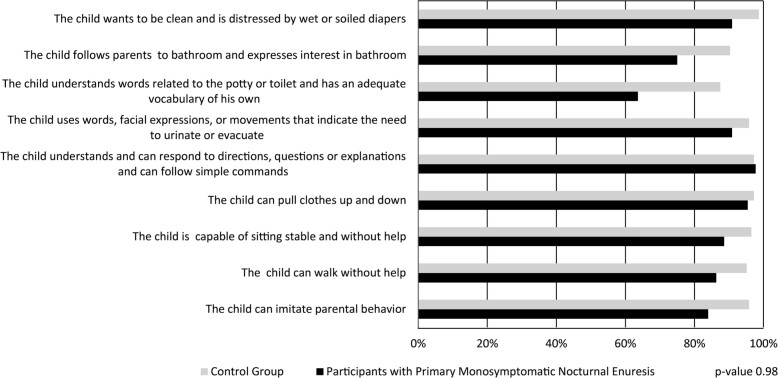
Main readiness signs described by parents/caregivers

Readiness signs were reported in 42.7% (44/103) of children and adolescents with PMNE and in 54.1% of the CG (144/266). These signs were absent in 40.8% (42/103) of the PMNE patients and 15% (40/266) of the CG. Only 16.5% (17/103) of the parents of PMNE cases did not remember if there were signs of readiness, while, for the parents of controls, the percentage was significantly higher reaching 31% (82/266) (p=0.001, Table-1). The main signs of readiness reported by parents are described in Figure-1. No differences were found in cases and controls (p=0.98).

The prevalence of the enuretic group and controls trained by the Child-Oriented approach was 87.4% (90/103) and 94% (250/266), respectively (OR= 0.44, 95% CI 0.21 a 0.94) (p = 0.039, Table-1).

There was no difference between the groups when evaluating the type of equipment used during the TT (p=0.99). Few cases and controls used a toilet with a seat reducer, footrest, or both. All participants in this study used disposable diapers during the TT. The type of equipment is described in Table-1.

Participants with PMNE started the daytime TT at 18.6 ± 8.7 months and those without PMNE at 17.4 ± 4.9 months (p=0.10). The average age of acquisition of daytime continence was 23.1 ± 11.3 months for cases and 21.4 ± 6.1 months for controls (p=0.06). Nocturnal TT's onset age was 22.3 ± 3.1 months in cases and 20.1 ± 5.5 months in the CG (p=0.08). The mean age of acquisition of nocturnal continence in controls was 27.34 ± 9.23 months (Table-1).

## DISCUSSION

We started our investigation by assessing whether parents identified specific skills to initiate the TT process in their children named readiness signs. These signs comprise three pillars of the child's neuropsychomotor development: physiological maturation, external feedback, and development of self-esteem and motivation (13, 20). Our study showed that parents of children and adolescents with PMNE remembered more signs of readiness than the parents of CG. A possible explanation would be that the parents of children and adolescents with PMNE usually try to identify the cause of enuresis. In contrast, the parents of healthy controls do not have the same motivation. Usually, parents of children with a disease or condition remember more related facts, in this case, a son or daughter with PMNE (25). There was no difference in the main signs of readiness found in the two groups. The same signals found in this study were the most frequently reported in two recent reviews (19, 26). The authors reported approximately twenty-one readiness signs, but both reviews considered the absence of knowledge about which and how many readiness signs are needed to start the TT (19, 26). On the other hand, we emphasize that the absence of signs of readiness was more frequent in participants with PMNE than in the CG, which may corroborate the Child-Oriented approach as a protective factor for PMNE occurrence in our series described below.

There is no consensus on the best method to be used for TT. The chosen approach depends on cultural differences, parental preferences, and expectations (13, 19). The approach should be individualized based on how the child learns best and the family's needs (13). In our study, children trained with the Child-Oriented approach had significantly less chance of exhibiting PMNE. Two studies showed an increase in the prevalence of PMNE, in children who received a threatening method of TT (27, 28). In one of them, PMNE was found 2.24 times more often in children submitted to a coercive and threatening approach (28). A recent study in Indonesia showed that if the TT quality is not good enough, the risk of enuresis is increased by 5.4 times compared to properly trained children. (29). In sharp contrast, Hackett et al. (30) showed an association between relaxed parental attitudes during TT with the occurrence of enuresis. However, the literature is very scarce regarding TT approaches and the occurrence of enuresis.

Before starting the TT process, parents must decide which equipment to use, usually the potty or toilet. The potty is preferred in the early stages because it is safe. Children feel more comfortable, do not need foot support or a seat reducer, and can be moved to other environments. The potty also offers the best biomechanical position for the child. Although the potty is preferred, some children like to imitate their parents and choose the toilet (10, 12, 20, 24). In the present sample, when evaluating the type of equipment used during the TT, about two third of children in both groups used the potty. The potty is the equipment indicated for approaching children and is considered a tool that helps assess readiness signs (13, 20). Notably, 24.9% of the enuresis group and 26.2% of the CG used the regular toilet without a seat reducer and/or footrest. It is important to reinforce that incorrect posture when urinating or defecating may lead to bowel bladder dysfunction (BBD), including enuresis (24). Although we did not find any association between the equipment used and the occurrence of enuresis, we suggest that children always use a toilet with a seat reducer and footrest or potty. The proper equipment results in a feeling of safety and a more physiological position to facilitate evacuation and urination and prevent BBD. We did not find in the literature studies that evaluated the relationship between the TT equipment and the presence of enuresis.

In our series, PMNE children and adolescents started daytime TT at 18.6 months and nighttime TT at 22.3 months, achieving daytime continence at 23.1 months, therefore earlier than recommended. Most studies suggested the beginning of TT between 24 and 36 months (13, 15) and showed that the age for staying dry during the day would be 32.5 to 35 months (18) and for complete acquisition of continence around 36 to 39 months (13, 15). Despite this, we found no statistical difference between cases and controls. The literature is still controversial about the relationship between the age at the beginning and end of the TT and its association with PMNE. The early onset of nocturnal TT (<30 months) was associated with early nocturnal continence and a lower rate of enuresis (31). Acikgoz et al. (11) reported a relationship between urinary incontinence only during the day, monosymptomatic and non-monosymptomatic enuresis, and initiation of the TT process after one year of age. In this regard, Akis et al. (32) showed that if the age of TT was late (> 24 months), the risk of enuresis was 3.04 times higher than in controls. Tokar et al. (33) showed an association between enuresis and age at the beginning of TT. They reported a higher reported likelihood of PMNE in children who began TT at four to six years or later. On the other hand, one recent study described that the onset of TT training after 24 months was not associated with isolated enuresis but strongly related to isolated daytime urinary incontinence, delayed bladder control, and school-age urinary incontinence (34).

We are aware of the limitations of this study. First, recall bias concerning TT data must be considered, especially in the control group. Caregivers of children with a disease or condition tend to be more accurate in describing it, and the opposite may occur in the CG (25). A second point concerns the TT. The assessment of the TT is challenging due to heterogeneity and methodological flaws, including bias, lack of standardization, differences in terminology and cultural definitions of successes and failures. Finally, this study has a relatively small sample size that precludes the detection of statistical differences, as for instance, regarding gender.

However, this study shows the importance for the healthcare team and family members to discuss the TT process. In general, healthcare professionals are only sought out for advice on TT when problems occur. The role of the healthcare team in the TT process is multifaceted. It includes the assessment of the child's signs of readiness, the investigation of family dynamics, the development of short- and long-term follow-up goals, and the identification of risk factors for failure.

In conclusion, the age of onset of TT and acquisition of daytime continence and the type of equipment were not associated with a higher occurrence of PMNE. On the other hand, parents of the children and adolescent with PMNE remembered more frequently of the signs of readiness than parents of the controls. The Child-Oriented approach was a protective factor for the occurrence of PMNE.

## ABBREVIATIONS

ICCS -: International Children's Continence Society
PMNE -: Primary monosymptomatic nocturnal enuresis
TT -: Toilet training
AAP -: American Academy of Pediatrics
CG -: Control Group
DVSS -: Dysfunctional Voiding Score Symptom
LUTS -: Lower urinary tract symptoms
OR -: Odds ratio
BBD -: Bowel bladder dysfunction
